# [μ-Bis(trimethyl­silyl)amido]bis­[μ-*N*,*N*-dimethyl-*N*′,*N*′′-bis­(trimethyl­silyl)guanidinato]-*triangulo*-tricopper(I)

**DOI:** 10.1107/S1600536809008745

**Published:** 2009-03-14

**Authors:** Donglong Guo, Xiaoli Qiao, Hong-Bo Tong, Meisu Zhou

**Affiliations:** a School of Life Science and Technology, Shanxi University, Taiyuan 030006, People’s Republic of China; b Institute of Applied Chemistry, Shanxi University, Taiyuan 030006, People’s Republic of China

## Abstract

The title compound, [Cu_3_(C_6_H_18_NSi_2_)(C_9_H_24_N_3_Si_2_)_2_], is a trinuclear Cu^I^ complex. A crystallographic twofold axis passes through one Cu^I^ atom and the N atom of the bis­(trimethyl­silyl)amide ligand that bridges between the other two Cu^I^ atoms. The Cu—Cu bonds bridged by the guanadinate ligands [2.7913 (9) Å] are slightly longer than the Cu—Cu bond bridged by the bis­(trimethyl­silyl)amide ligand [2.6405 (11) Å].

## Related literature

For background literature concerning the coordination chemistry of guanidinates, see: Chandra *et al.* (1970 ▶); Barker & Kilner (1994 ▶); Edelmann (1994 ▶); Bailey & Pace (2001 ▶); Zhou *et al.* (2007 ▶).
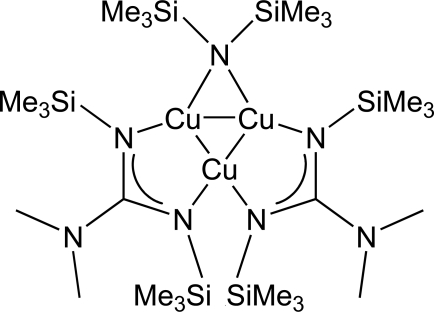


## Experimental

### 

#### Crystal data

[Cu_3_(C_6_H_18_NSi_2_)(C_9_H_24_N_3_Si_2_)_2_]
*M* *_r_* = 812.00
Monoclinic, 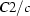

*a* = 16.445 (3) Å
*b* = 18.653 (4) Å
*c* = 14.046 (3) Å
β = 96.943 (3)°
*V* = 4277.1 (15) Å^3^
*Z* = 4
Mo *K*α radiation
μ = 1.67 mm^−1^
*T* = 213 K
0.30 × 0.20 × 0.20 mm

#### Data collection

Siemens SMART CCD diffractometer
Absorption correction: multi-scan (*SADABS*; Sheldrick, 1997 ▶) *T*_min_ = 0.622, *T*_max_ = 0.717
8714 measured reflections
3773 independent reflections
3394 reflections with *I* > 2σ(*I*)
*R*_int_ = 0.033

#### Refinement

*R*[*F* ^2^ > 2σ(*F* ^2^)] = 0.056
*wR*(*F* ^2^) = 0.118
*S* = 1.26
3773 reflections
193 parameters
H-atom parameters constrained
Δρ_max_ = 0.57 e Å^−3^
Δρ_min_ = −0.46 e Å^−3^

### 

Data collection: *SMART* (Siemens, 1996 ▶); cell refinement: *SAINT* (Siemens, 1996 ▶); data reduction: *SAINT*; program(s) used to solve structure: *SHELXS97* (Sheldrick, 2008 ▶); program(s) used to refine structure: *SHELXL97* (Sheldrick, 2008 ▶); molecular graphics: *ORTEP-3* (Farrugia, 1997 ▶); software used to prepare material for publication: *SHELXL97*.

## Supplementary Material

Crystal structure: contains datablocks I, global. DOI: 10.1107/S1600536809008745/bi2357sup1.cif

Structure factors: contains datablocks I. DOI: 10.1107/S1600536809008745/bi2357Isup2.hkl

Additional supplementary materials:  crystallographic information; 3D view; checkCIF report

## Figures and Tables

**Fig. 1.**
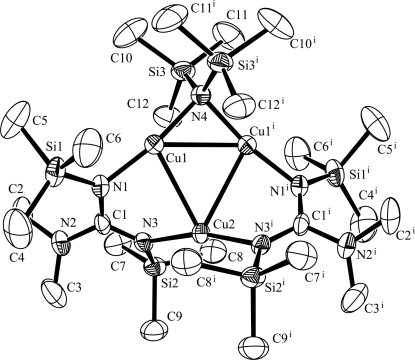
Molecular structure showing displacement ellipsoids at 50% probability. H atoms are omitted. Symmetry code: (i) -x, y, 3/2 - z.

## supplementary crystallographic information

### Comment

Since the first guanidinato complex was reported by Lapper and coworkers (Chandra *et al.*, 1970), the coordination chemistry of guanidinates has been well explored for main group metals as well as transition metals (Bailey & Pace, 2001; Barker & Kilner, 1994; 1994; Edelmann, 1994). The trigonal-planar CN_3_ unit provides easy accessibillity and the possibility of substituent variation, which allows for tuning of the steric and electronic properties of the ligands. Recently, we reported a series of early transition metal guanidinates and their applications in the polymerization of ethylene (Zhou *et al.*, 2007). Here we describe the synthesis and crystal structure of a new copper(I) guanidinato complex.

The molecular structure is illustrated in Fig. 1. In the trinuclear copper compound, each Cu^I^ atom coordinates to the other two Cu^I^ atoms and two N from the ligands. Atoms Cu1, Cu2, Cu1^i^ and N4 are exactly co-planar with a crystallographic 2-fold rotation axis passing through Cu2 and N4. The bond lengths Cu1—Cu2 and Cu1^i^—Cu2 are therefore identical, whereas the bond length Cu1—Cu1^i^ is slightly shorter (Table 1). The Cu1—N1 and Cu2—N3 bond lengths are 1.875 (3) and 1.885 (3) Å, respectively. In the guanidinato ligand, the bond lengths C1—N1, C1—N2 and C1—N3 are 1.329 (5), 1.386 (5) and 1.341 (5) Å, respectively. The bond angle N1—C1—N3 is 122.8 (3)°. The dihedral angle between N1/C1/N3 and Cu1/Cu2/N3 is 31.8° and that between Cu1/Cu2/Cu1^i^ and Cu1/Cu2/N3 is 42.0°.

### Experimental

(CH_3_)_2_NCN (0.22 ml, 2.76 mmol) was added to a solution of LiN(SiMe_3_)_2_ (0.46 g, 2.76 mmol) in THF (30 ml) at -78°C. The resulting mixture was warmed to room temperature and stirred for 2 h. CuCl (0.27 g,2.76 mmol) was the added at -78°C and the mixture was warmed to again to room temperature and stirred for 24 h. The volatiles were removed in *vacuo* and the residue was extracted with dichloromethane then filtered. The filtrate was concentrated to give colorless crystals (0.14 g, 19%). M.p.: 398–400 K. ^1^H NMR (CDCl_3_):δ 0.10–0.43 (m, 54H, SiMe_3_), 2.86 (m, 12H, N(C*H*_3_)_2_). ^13^C NMR (CDCl_3_): δ 1.74~7.44 (SiMe_3_), 42.16 (N(*C*H_3_)_2_), 172.8 (N*C*N).

### Refinement

H atoms of the methyl groups were placed geometrically with C—H = 0.97 Å and allowed to ride during subsequent refinement with *U*_iso_(H) = 1.5*U*_eq_(C).

### Crystal data

**Table:**

[Cu_3_(C_6_H_18_NSi_2_)(C_9_H_24_N_3_Si_2_)_2_]	*F*(000) = 1720
*M**_r_* = 812.00	*D*_x_ = 1.261 Mg m^−^^3^
Monoclinic, *C*2/*c*	Mo *K*α radiation, λ = 0.71073 Å
Hall symbol: -C 2yc	Cell parameters from 3322 reflections
*a* = 16.445 (3) Å	θ = 2.3–27.5°
*b* = 18.653 (4) Å	µ = 1.67 mm^−^^1^
*c* = 14.046 (3) Å	*T* = 213 K
β = 96.943 (3)°	Block, colourless
*V* = 4277.1 (15) Å^3^	0.30 × 0.20 × 0.20 mm
*Z* = 4	

### Data collection

**Table:**

Siemens SMART CCD diffractometer	3773 independent reflections
Radiation source: fine-focus sealed tube	3394 reflections with *I* > 2σ(*I*)
graphite	*R*_int_ = 0.033
ω scans	θ_max_ = 25.0°, θ_min_ = 1.7°
Absorption correction: multi-scan (*SADABS*; Sheldrick, 1997)	*h* = −19→16
*T*_min_ = 0.622, *T*_max_ = 0.717	*k* = −21→22
8714 measured reflections	*l* = −16→15

### Refinement

**Table:**

Refinement on *F*^2^	Primary atom site location: structure-invariant direct methods
Least-squares matrix: full	Secondary atom site location: difference Fourier map
*R*[*F*^2^ > 2σ(*F*^2^)] = 0.056	Hydrogen site location: inferred from neighbouring sites
*wR*(*F*^2^) = 0.118	H-atom parameters constrained
*S* = 1.26	*w* = 1/[σ^2^(*F*_o_^2^) + (0.0417*P*)^2^ + 4.1466*P*] where *P* = (*F*_o_^2^ + 2*F*_c_^2^)/3
3773 reflections	(Δ/σ)_max_ < 0.001
193 parameters	Δρ_max_ = 0.57 e Å^−^^3^
0 restraints	Δρ_min_ = −0.46 e Å^−^^3^

### Special details

**Table:**

Geometry. All e.s.d.'s (except the e.s.d. in the dihedral angle between two l.s. planes) are estimated using the full covariance matrix. The cell e.s.d.'s are taken into account individually in the estimation of e.s.d.'s in distances, angles and torsion angles; correlations between e.s.d.'s in cell parameters are only used when they are defined by crystal symmetry. An approximate (isotropic) treatment of cell e.s.d.'s is used for estimating e.s.d.'s involving l.s. planes.
Refinement. Refinement of *F*^2^ against ALL reflections. The weighted *R*-factor *wR* and goodness of fit *S* are based on *F*^2^, conventional *R*-factors *R* are based on *F*, with *F* set to zero for negative *F*^2^. The threshold expression of *F*^2^ > σ(*F*^2^) is used only for calculating *R*-factors(gt) *etc*. and is not relevant to the choice of reflections for refinement. *R*-factors based on *F*^2^ are statistically about twice as large as those based on *F*, and *R*- factors based on ALL data will be even larger.

### Fractional atomic coordinates and isotropic or equivalent isotropic displacement parameters (Å^2^)

**Table:**

	*x*	*y*	*z*	*U*_iso_*/*U*_eq_	
Cu1	0.00565 (3)	0.28899 (3)	0.65704 (4)	0.03181 (17)	
Cu2	0.0000	0.15714 (4)	0.7500	0.0297 (2)	
N1	0.0253 (2)	0.22736 (18)	0.5568 (2)	0.0335 (8)	
N2	0.1160 (3)	0.1407 (2)	0.5116 (3)	0.0489 (11)	
N3	0.0812 (2)	0.14177 (18)	0.6683 (3)	0.0327 (8)	
N4	0.0000	0.3629 (2)	0.7500	0.0331 (12)	
Si1	−0.03059 (9)	0.24764 (8)	0.44721 (9)	0.0484 (4)	
Si2	0.16644 (8)	0.10002 (7)	0.73019 (10)	0.0384 (3)	
Si3	0.09487 (9)	0.40530 (7)	0.75808 (11)	0.0444 (4)	
C1	0.0727 (3)	0.1708 (2)	0.5803 (3)	0.0326 (10)	
C2	0.1565 (4)	0.1842 (3)	0.4465 (4)	0.0711 (18)	
H2A	0.1295	0.1780	0.3817	0.107*	
H2B	0.2134	0.1697	0.4493	0.107*	
H2C	0.1538	0.2342	0.4648	0.107*	
C3	0.1235 (4)	0.0641 (3)	0.5000 (4)	0.077 (2)	
H3A	0.0899	0.0397	0.5419	0.116*	
H3B	0.1803	0.0501	0.5163	0.116*	
H3C	0.1055	0.0512	0.4339	0.116*	
C4	−0.0495 (4)	0.1674 (4)	0.3693 (4)	0.085 (2)	
H4A	0.0018	0.1512	0.3490	0.127*	
H4B	−0.0875	0.1796	0.3133	0.127*	
H4C	−0.0726	0.1294	0.4048	0.127*	
C5	0.0166 (4)	0.3231 (4)	0.3864 (5)	0.087 (2)	
H5A	0.0322	0.3609	0.4325	0.131*	
H5B	−0.0226	0.3417	0.3352	0.131*	
H5C	0.0648	0.3060	0.3598	0.131*	
C6	−0.1333 (3)	0.2798 (4)	0.4694 (4)	0.0747 (19)	
H6A	−0.1633	0.2407	0.4946	0.112*	
H6B	−0.1629	0.2966	0.4097	0.112*	
H6C	−0.1273	0.3187	0.5155	0.112*	
C7	0.2640 (3)	0.1337 (3)	0.6908 (4)	0.0577 (15)	
H7A	0.2668	0.1196	0.6248	0.087*	
H7B	0.3102	0.1133	0.7315	0.087*	
H7C	0.2658	0.1855	0.6957	0.087*	
C8	0.1675 (3)	0.1227 (3)	0.8591 (4)	0.0543 (14)	
H8A	0.1733	0.1742	0.8675	0.081*	
H8B	0.2131	0.0987	0.8962	0.081*	
H8C	0.1165	0.1072	0.8809	0.081*	
C9	0.1591 (4)	0.0000 (2)	0.7255 (4)	0.0586 (15)	
H9A	0.1034	−0.0146	0.7321	0.088*	
H9B	0.1962	−0.0205	0.7774	0.088*	
H9C	0.1738	−0.0169	0.6646	0.088*	
C10	0.1033 (4)	0.4625 (3)	0.6504 (5)	0.085 (2)	
H10A	0.0660	0.5027	0.6504	0.128*	
H10B	0.0894	0.4343	0.5927	0.128*	
H10C	0.1590	0.4801	0.6523	0.128*	
C11	0.1138 (4)	0.4635 (3)	0.8661 (5)	0.080 (2)	
H11A	0.0990	0.4379	0.9216	0.120*	
H11B	0.0808	0.5067	0.8564	0.120*	
H11C	0.1713	0.4765	0.8767	0.120*	
C12	0.1783 (3)	0.3378 (3)	0.7637 (4)	0.0574 (15)	
H12A	0.2309	0.3620	0.7708	0.086*	
H12B	0.1725	0.3097	0.7052	0.086*	
H12C	0.1751	0.3064	0.8182	0.086*	

### Atomic displacement parameters (Å^2^)

**Table:**

	*U*^11^	*U*^22^	*U*^33^	*U*^12^	*U*^13^	*U*^23^
Cu1	0.0350 (3)	0.0301 (3)	0.0309 (3)	0.0019 (2)	0.0064 (2)	0.0008 (2)
Cu2	0.0272 (4)	0.0326 (4)	0.0308 (4)	0.000	0.0095 (3)	0.000
N1	0.037 (2)	0.040 (2)	0.0247 (19)	0.0004 (18)	0.0059 (15)	−0.0007 (16)
N2	0.058 (3)	0.053 (3)	0.040 (2)	0.002 (2)	0.025 (2)	−0.0066 (19)
N3	0.032 (2)	0.0316 (19)	0.037 (2)	0.0019 (16)	0.0125 (16)	−0.0031 (16)
N4	0.035 (3)	0.031 (3)	0.035 (3)	0.000	0.007 (2)	0.000
Si1	0.0512 (9)	0.0670 (10)	0.0269 (7)	−0.0083 (7)	0.0038 (6)	0.0050 (6)
Si2	0.0318 (7)	0.0326 (7)	0.0523 (8)	0.0043 (6)	0.0111 (6)	0.0007 (6)
Si3	0.0451 (8)	0.0317 (7)	0.0583 (9)	−0.0068 (6)	0.0139 (7)	−0.0011 (6)
C1	0.032 (3)	0.039 (3)	0.029 (2)	−0.008 (2)	0.0146 (19)	−0.0080 (19)
C2	0.065 (4)	0.104 (5)	0.051 (4)	0.006 (4)	0.033 (3)	0.002 (3)
C3	0.106 (5)	0.069 (4)	0.060 (4)	0.018 (4)	0.024 (4)	−0.023 (3)
C4	0.082 (5)	0.118 (6)	0.052 (4)	−0.026 (4)	−0.003 (3)	−0.024 (4)
C5	0.092 (5)	0.107 (5)	0.062 (4)	−0.017 (4)	0.005 (4)	0.042 (4)
C6	0.061 (4)	0.107 (5)	0.054 (4)	0.014 (4)	−0.004 (3)	0.017 (3)
C7	0.035 (3)	0.056 (3)	0.085 (4)	0.002 (3)	0.020 (3)	0.007 (3)
C8	0.048 (3)	0.053 (3)	0.059 (3)	0.007 (3)	−0.003 (3)	0.004 (3)
C9	0.063 (4)	0.038 (3)	0.077 (4)	0.007 (3)	0.019 (3)	0.005 (3)
C10	0.074 (5)	0.074 (4)	0.109 (6)	−0.022 (4)	0.017 (4)	0.041 (4)
C11	0.074 (4)	0.064 (4)	0.105 (5)	−0.029 (3)	0.021 (4)	−0.042 (4)
C12	0.041 (3)	0.046 (3)	0.086 (4)	−0.008 (2)	0.013 (3)	−0.010 (3)

### Geometric parameters (Å, °)

**Table:**

Cu1—Cu1^i^	2.6405 (11)	C3—H3B	0.970
Cu1—Cu2	2.7913 (9)	C3—H3C	0.970
Cu2—Cu1^i^	2.7912 (9)	C4—H4A	0.970
Cu1—N1	1.875 (3)	C4—H4B	0.970
Cu1—N4	1.908 (3)	C4—H4C	0.970
Cu2—N3	1.885 (3)	C5—H5A	0.970
Cu2—N3^i^	1.885 (3)	C5—H5B	0.970
N1—C1	1.329 (5)	C5—H5C	0.970
N1—Si1	1.737 (4)	C6—H6A	0.970
N2—C1	1.386 (5)	C6—H6B	0.970
N2—C3	1.444 (6)	C6—H6C	0.970
N2—C2	1.445 (6)	C7—H7A	0.970
N3—C1	1.341 (5)	C7—H7B	0.970
N3—Si2	1.742 (4)	C7—H7C	0.970
N4—Si3	1.741 (3)	C8—H8A	0.970
N4—Si3^i^	1.741 (3)	C8—H8B	0.970
N4—Cu1^i^	1.909 (3)	C8—H8C	0.970
Si1—C6	1.853 (6)	C9—H9A	0.970
Si1—C4	1.859 (6)	C9—H9B	0.970
Si1—C5	1.865 (6)	C9—H9C	0.970
Si2—C8	1.858 (5)	C10—H10A	0.970
Si2—C7	1.868 (5)	C10—H10B	0.970
Si2—C9	1.871 (5)	C10—H10C	0.970
Si3—C12	1.856 (5)	C11—H11A	0.970
Si3—C11	1.862 (6)	C11—H11B	0.970
Si3—C10	1.870 (6)	C11—H11C	0.970
C2—H2A	0.970	C12—H12A	0.970
C2—H2B	0.970	C12—H12B	0.970
C2—H2C	0.970	C12—H12C	0.970
C3—H3A	0.970		
			
N1—Cu1—N4	169.49 (13)	N2—C3—H3C	109.5
N1—Cu1—Cu1^i^	141.39 (11)	H3A—C3—H3C	109.5
N4—Cu1—Cu1^i^	46.23 (10)	H3B—C3—H3C	109.5
N1—Cu1—Cu2	80.18 (11)	Si1—C4—H4A	109.5
N4—Cu1—Cu2	108.00 (10)	Si1—C4—H4B	109.5
Cu1^i^—Cu1—Cu2	61.768 (14)	H4A—C4—H4B	109.5
N3—Cu2—N3^i^	162.5 (2)	Si1—C4—H4C	109.5
N3—Cu2—Cu1^i^	119.01 (11)	H4A—C4—H4C	109.5
N3^i^—Cu2—Cu1^i^	77.47 (10)	H4B—C4—H4C	109.5
N3—Cu2—Cu1	77.47 (10)	Si1—C5—H5A	109.5
N3^i^—Cu2—Cu1	119.02 (11)	Si1—C5—H5B	109.5
Cu1^i^—Cu2—Cu1	56.46 (3)	H5A—C5—H5B	109.5
C1—N1—Si1	128.6 (3)	Si1—C5—H5C	109.5
C1—N1—Cu1	116.7 (3)	H5A—C5—H5C	109.5
Si1—N1—Cu1	114.3 (2)	H5B—C5—H5C	109.5
C1—N2—C3	122.5 (4)	Si1—C6—H6A	109.5
C1—N2—C2	121.9 (4)	Si1—C6—H6B	109.5
C3—N2—C2	115.6 (4)	H6A—C6—H6B	109.5
C1—N3—Si2	129.0 (3)	Si1—C6—H6C	109.5
C1—N3—Cu2	119.8 (3)	H6A—C6—H6C	109.5
Si2—N3—Cu2	110.53 (19)	H6B—C6—H6C	109.5
Si3—N4—Si3^i^	125.9 (3)	Si2—C7—H7A	109.5
Si3—N4—Cu1	104.80 (7)	Si2—C7—H7B	109.5
Si3^i^—N4—Cu1	113.64 (8)	H7A—C7—H7B	109.5
Si3—N4—Cu1^i^	113.64 (8)	Si2—C7—H7C	109.5
Si3^i^—N4—Cu1^i^	104.80 (7)	H7A—C7—H7C	109.5
Cu1—N4—Cu1^i^	87.5 (2)	H7B—C7—H7C	109.5
N1—Si1—C6	108.5 (2)	Si2—C8—H8A	109.5
N1—Si1—C4	112.3 (3)	Si2—C8—H8B	109.5
C6—Si1—C4	105.6 (3)	H8A—C8—H8B	109.5
N1—Si1—C5	111.4 (3)	Si2—C8—H8C	109.5
C6—Si1—C5	105.7 (3)	H8A—C8—H8C	109.5
C4—Si1—C5	112.8 (3)	H8B—C8—H8C	109.5
N3—Si2—C8	107.3 (2)	Si2—C9—H9A	109.5
N3—Si2—C7	111.7 (2)	Si2—C9—H9B	109.5
C8—Si2—C7	107.8 (3)	H9A—C9—H9B	109.5
N3—Si2—C9	112.6 (2)	Si2—C9—H9C	109.5
C8—Si2—C9	104.8 (2)	H9A—C9—H9C	109.5
C7—Si2—C9	112.3 (2)	H9B—C9—H9C	109.5
N4—Si3—C12	110.3 (2)	Si3—C10—H10A	109.5
N4—Si3—C11	112.2 (2)	Si3—C10—H10B	109.5
C12—Si3—C11	108.1 (3)	H10A—C10—H10B	109.5
N4—Si3—C10	111.1 (2)	Si3—C10—H10C	109.5
C12—Si3—C10	107.1 (3)	H10A—C10—H10C	109.5
C11—Si3—C10	107.7 (3)	H10B—C10—H10C	109.5
N1—C1—N3	122.8 (3)	Si3—C11—H11A	109.5
N1—C1—N2	119.0 (4)	Si3—C11—H11B	109.5
N3—C1—N2	118.2 (4)	H11A—C11—H11B	109.5
N2—C2—H2A	109.5	Si3—C11—H11C	109.5
N2—C2—H2B	109.5	H11A—C11—H11C	109.5
H2A—C2—H2B	109.5	H11B—C11—H11C	109.5
N2—C2—H2C	109.5	Si3—C12—H12A	109.5
H2A—C2—H2C	109.5	Si3—C12—H12B	109.5
H2B—C2—H2C	109.5	H12A—C12—H12B	109.5
N2—C3—H3A	109.5	Si3—C12—H12C	109.5
N2—C3—H3B	109.5	H12A—C12—H12C	109.5
H3A—C3—H3B	109.5	H12B—C12—H12C	109.5

Symmetry codes: (i) −*x*, *y*, −*z*+3/2.
